# Supplementary material for: Enhanced carbonyl stress induces irreversible multimerization of CRMP2 in schizophrenia pathogenesis
Source: Life Sci Alliance. 2019 Oct 7;2(5):e201900478. doi: 10.26508/lsa.201900478 (PMC6781483; doi:10.26508/lsa.201900478)
Supplement: Supplementary file 1 [file LSA-2019-00478_SdataFS1.pdf]

Figure S1 Toyoshima & Jiang et al.

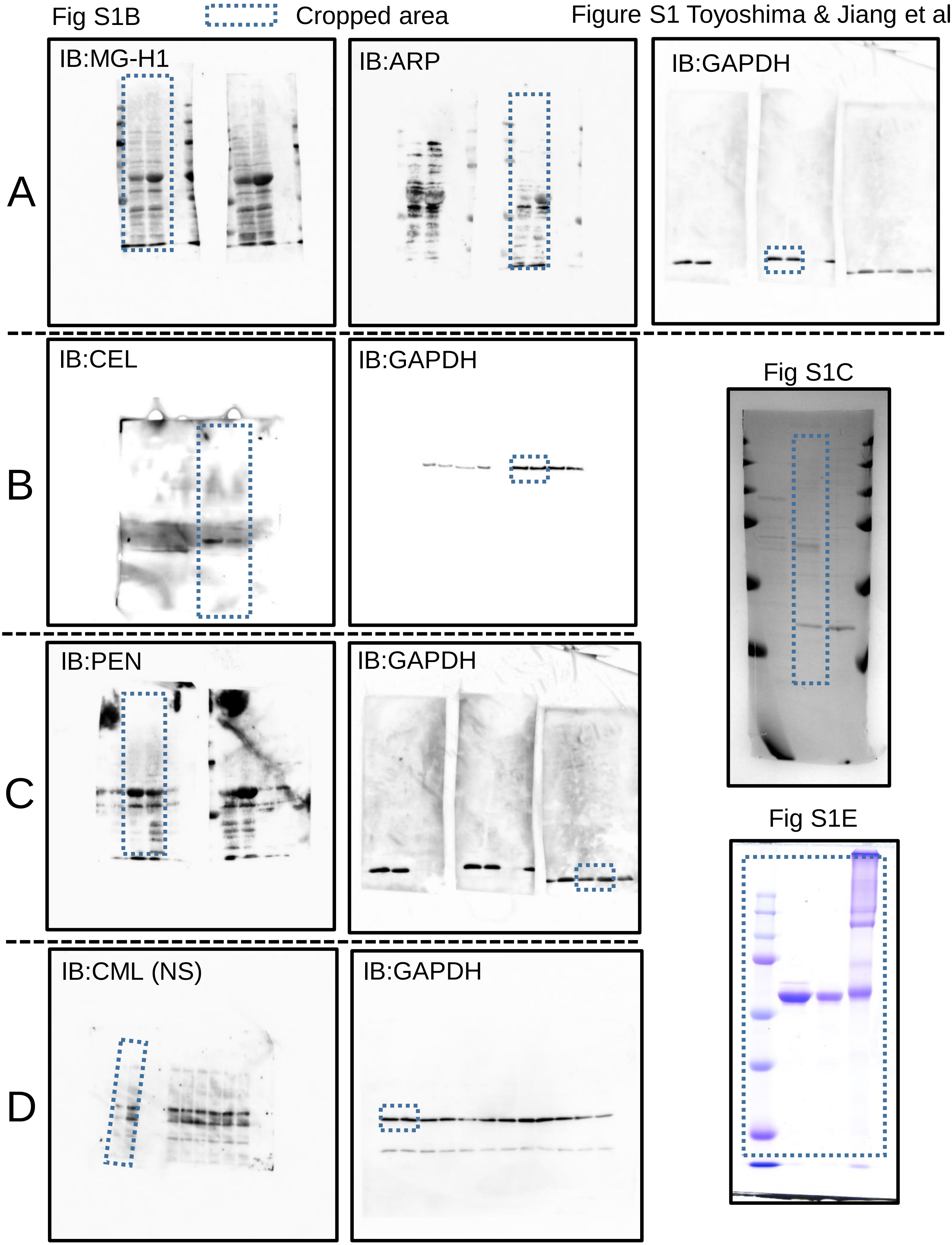

To exclude the experimental error in sample application, the same membranes were blotted several times with depriving process for IB shown in respective panels (A, B, C and D).
