# Supplementary figures and images for: Enhanced carbonyl stress induces irreversible multimerization of CRMP2 in schizophrenia pathogenesis
Source: Life Sci Alliance. 2019 Oct 7;2(5):e201900478. doi: 10.26508/lsa.201900478 (PMC6781483; doi:10.26508/lsa.201900478)

Fig 1C

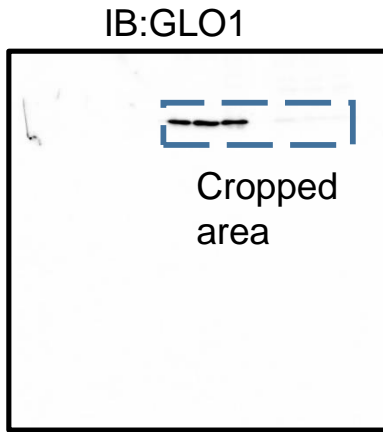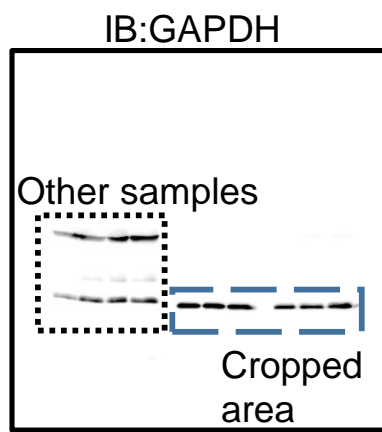

Fig 1I

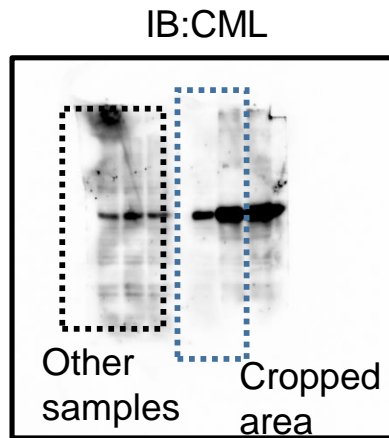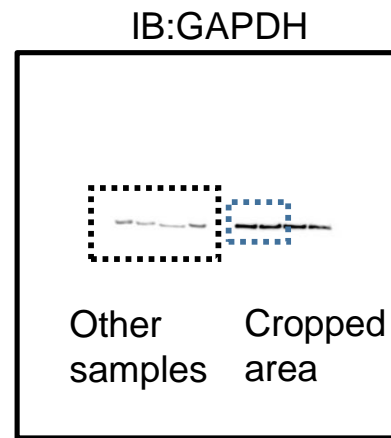

Fig 1J

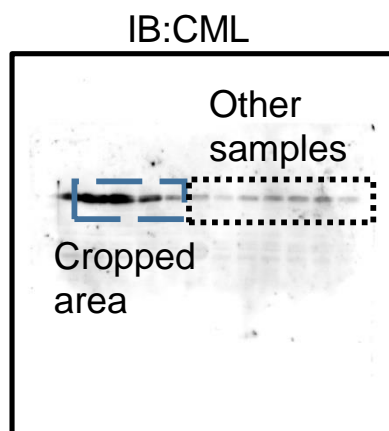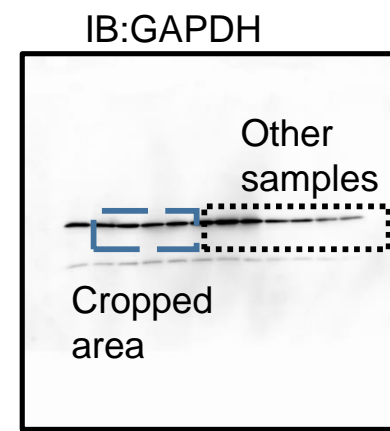

Fig 1L

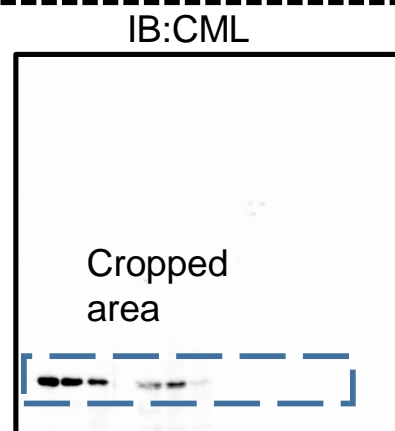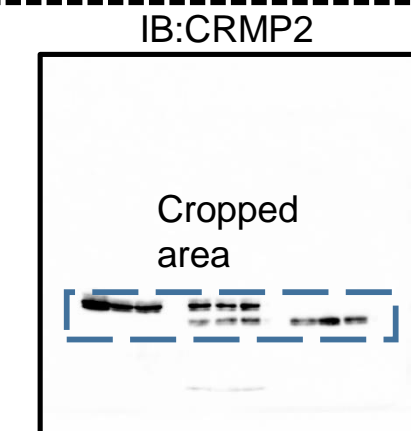

Supplement: Supplementary file 2 [file LSA-2019-00478_SdataF1.pdf]

Cropped area

Fig 3C

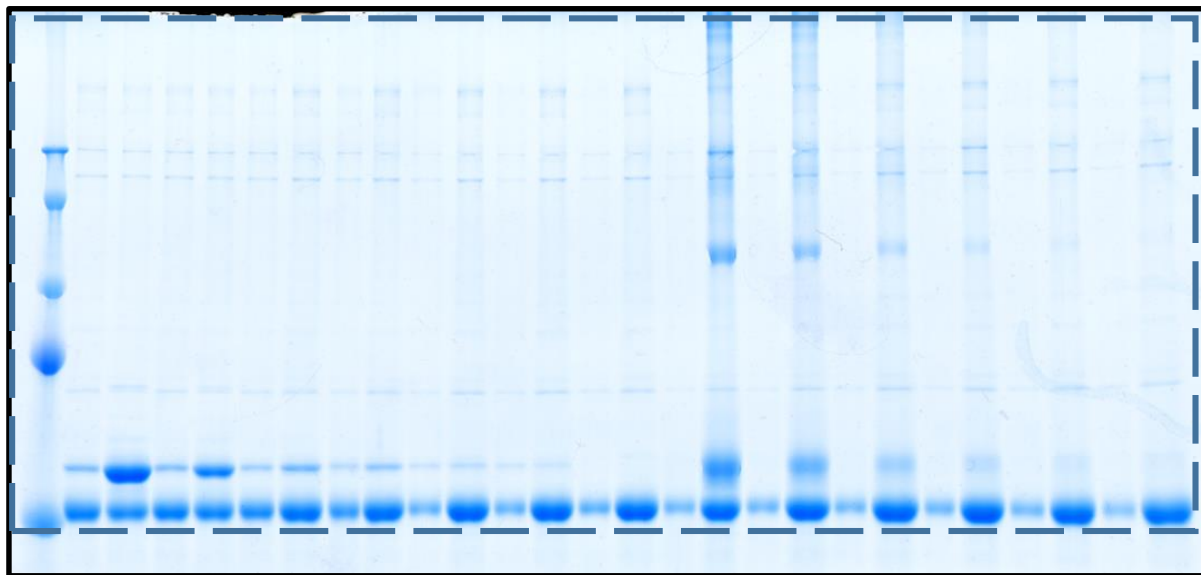

Figure 3 Toyoshima & Jiang et al.

Supplement: Supplementary file 3 [file LSA-2019-00478_SdataF3.pdf]

CRMP2

AGE-CRMP2

Fig S3A

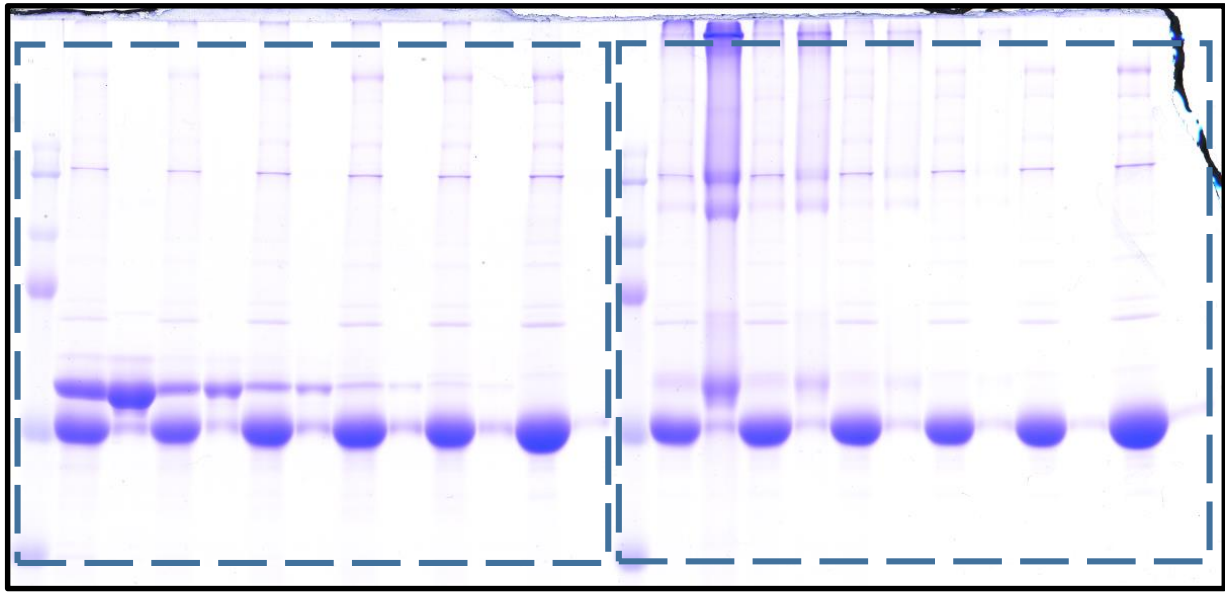

Cropped area

Cropped area

Figure S3 Toyoshima & Jiang et al.

Supplement: Supplementary file 4 [file LSA-2019-00478_SdataFS3.pdf]

CRMP2

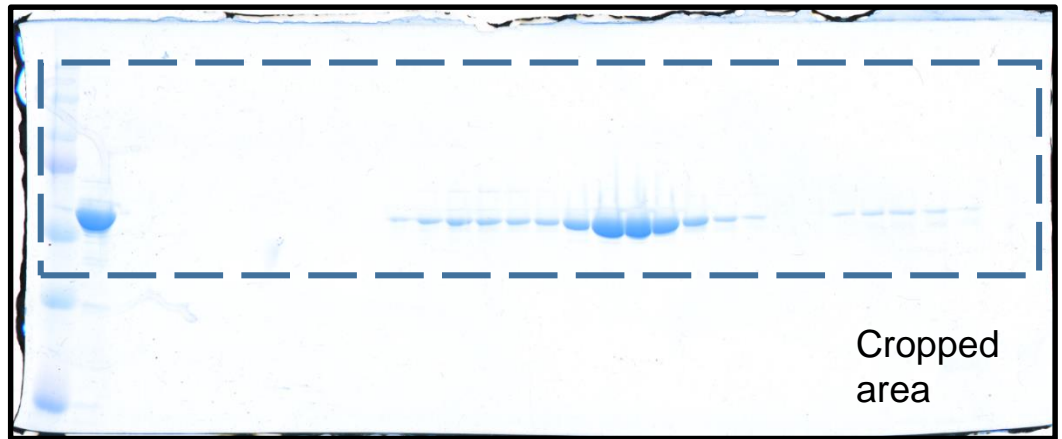

Fig 5B

AGE-CRMP2

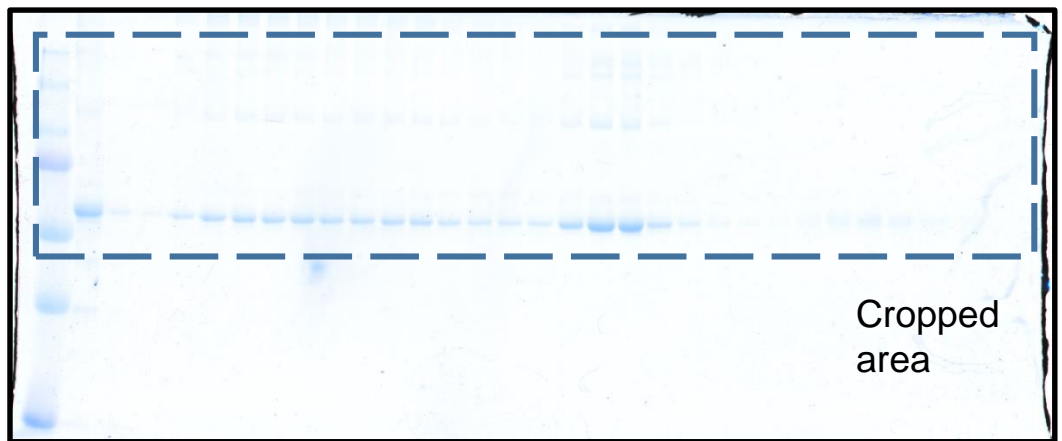

IB:Myc-tag

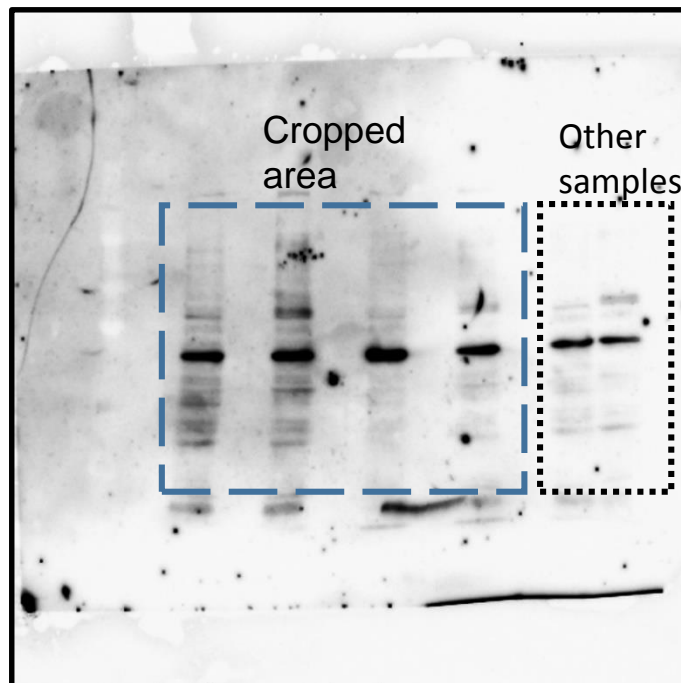

Fig 5C

Supplement: Supplementary file 5 [file LSA-2019-00478_SdataF5.pdf]
